# Bacterial Etiology and Antimicrobial Susceptibility Patterns in Pediatric Intra-Abdominal Infections—Implications for Empirical Treatment and Antimicrobial Stewardship

**DOI:** 10.3390/ph19071121

**Published:** 2026-07-20

**Authors:** Florin Daniel Enache, Ancuta Lupu, Tatiana Chisnoiu, Adriana Luminita Balasa, Emil Anton, Gabriel Florin Panculescu, Ioana Livia Suliman, Violeta Popovici, Ramona Mihaela Stoicescu, Iulian Manac, Vasile Valeriu Lupu, Cristina Maria Mihai

**Affiliations:** 1 Department of Pediatric Surgery and Orthopedics, Faculty of General Medicine, “Ovidius” University of Constanta, 900470 Constanta, Romania; florin.enache@365.univ-ovidius.ro (F.D.E.); iulian.manac@gmail.com (I.M.); 2 Pediatric Department, Faculty of General Medicine, “Ovidius” University of Constanta, 900470 Constanta, Romania; adriana.balasa@365.univ-ovidius.ro (A.L.B.); cristina_mihai@365.univ-ovidius.ro (C.M.M.); 3 “Grigore T. Popa” University of Medicine and Pharmacy, 700115 Iasi, Romania; ancuta.ignat1@umfiasi.ro (A.L.); emil.anton@umfiasi.ro (E.A.); vasile.lupu@umfiasi.ro (V.V.L.); 4 Pediatrics, County Clinical Emergency Hospital of Constanta, 900591 Constanta, Romania; 5 Faculty of General Medicine, “Ovidius” University of Constanta, 900470 Constanta, Romania; gabriel.panculescu@yahoo.ro (G.F.P.); panculescu_i@yahoo.com (I.L.S.); 6 Center for Mountain Economics, “Costin C. Kiritescu” National Institute of Economic Research (INCE-CEMONT), Romanian Academy, 725700 Vatra-Dornei, Romania; 7 Department of Microbiology and Immunology, Faculty of Pharmacy, “Ovidius” University of Constanta, Str. Căpitan Aviator Al. Șerbănescu, nr.6, Campus Corp C, 900470 Constanta, Romania; ramona.stoicescu@univ-ovidius.ro

**Keywords:** pediatric patients, intra-abdominal infections, surgical management, bacterial etiology, associated pathogens, antibiotic susceptibility

## Abstract

**Objectives**: Intra-abdominal infections (IAIs) requiring surgical intervention represent a significant cause of morbidity in pediatric patients, often leading to prolonged hospitalization and increased antimicrobial exposure. This study aimed to characterize the distribution of pathogenic bacteria, antimicrobial resistance patterns, and clinical associations in pediatric intra-abdominal infections complicated by surgical site involvement. **Methods**: A retrospective observational study was conducted on children aged 0–16 years, who underwent surgery for intra-abdominal infections with microbiological confirmation. Peritoneal fluid, pus, and other intraoperative or postoperative specimens were analyzed using standard microbiological techniques. Infections were classified as monomicrobial or polymicrobial, and antimicrobial susceptibility was assessed phenotypically. Associations between bacterial pathogens, patient age, underlying diagnosis, surgical procedures, and antibiotic susceptibility patterns were analyzed. **Results**: 177 pediatric patients were included. Appendicitis was the most common diagnosis (53.53%), followed by intra-abdominal abscesses (32.94%). Gram-negative (GN) bacteria predominated (44.07%), with *Escherichia coli* being the most frequently isolated pathogen (83.33%). Double and triple GN and GP associations were identified in approximately 25% of cases, particularly in abscesses, complicated appendicitis, and surgical site infections. Our findings revealed that piperacillin–tazobactam and carbapenems were expected to be effective against almost all GN pathogens identified in surgical specimens of pediatric patients. Pathogen distribution and antimicrobial susceptibility varied significantly according to age group and clinical diagnosis. **Conclusions**: In pediatric patients, intra-abdominal infections requiring surgical management were mainly caused by Gram-negative bacteria and polymicrobial associations. Effective treatment relies on prompt surgical source control and empiric broad-spectrum antimicrobial therapy, followed by culture-guided de-escalation to support antimicrobial stewardship.

## 1. Introduction

Intra-abdominal infections (IAIs) pose a significant healthcare challenge, contributing to high rates of morbidity and mortality among pediatric patients [1]. These infections are characterized by their heterogeneous nature, influenced by a variety of patient-specific factors, including underlying health conditions, infection severity, and individual responses to treatment. Due to this complexity, the successful management of IAI requires a comprehensive, multidisciplinary approach. Effectively treating IAI requires a collaborative effort from a diverse team of healthcare professionals. This team typically includes surgeons skilled in performing necessary surgical interventions, critical care physicians specializing in the management of critically ill patients, and infectious disease specialists who provide guidance on appropriate antimicrobial therapies. Additionally, pharmacists play a significant role in ensuring safe and effective medication management. The involvement of diagnostic and interventional radiologists is also vital, as they use advanced imaging techniques to guide diagnosis and treatment. Furthermore, nurses and advanced practice providers are essential in delivering bedside care, monitoring patient progress, and facilitating communication within the healthcare team.

Pediatric IAIs are mainly categorized as either uncomplicated or complicated (cIAI). An uncomplicated IAI affects a single infected abdominal organ without causing anatomical disruption [2]. At the same time, a cIAI involves the spread of infection into the peritoneal cavity, presenting as an abdominal abscess or diffuse peritonitis [3].

### 1.1. Clinical IAI Types in Pediatric Patients

Four distinct types are described, as follows:

Primary peritonitis—a microbial infection of the peritoneal fluid that occurs without gastrointestinal or other visceral perforation, abscess, or localized IAI. It rarely develops in healthy adults, but is more common in infancy and early childhood [4].

Secondary peritonitis—the most common type of cIAI, usually resulting from a breach in the integrity of the GI tract or infected organs. Common causes include penetrating or blunt abdominal trauma, appendicitis, biliary tract infections, and postoperative complications. In older pediatric patients, secondary peritonitis is mainly linked to complicated appendicitis, but it can also occur due to intussusception, incarcerated hernia, volvulus, or rupture of a Meckel’s diverticulum [5].

Necrotizing enterocolitis (NEC)—an inflammatory bowel necrosis that typically appears within the first 2 weeks of life and affects the terminal ileum; it is the leading cause of secondary peritonitis in neonates [6].

Intra-abdominal abscess—a loculated collection of pus within the peritoneal cavity or within a solid organ, commonly arising as a complication of perforated appendicitis or postoperative infections [7,8,9].

### 1.2. Epidemiology and Incidence

#### 1.2.1. Complicated Appendicitis

Acute appendicitis is the leading cause of community-acquired intra-abdominal infections (cIAIs) in children. A study of pediatric patients aged 1 month to 15 years found that most infections (92%, 113 of 123 cases) resulted from complicated appendicitis. Globally, acute appendicitis is a major cause of abdominal pain and the need for surgery, with 174–274 cases per 100,000 people annually, affecting both children and adults. When diagnosed, the rate of appendiceal perforation ranges from 20% to 35%. In children under 3 years old, perforation occurs in 10–20% of cases, while in those aged 10–17 years, it also occurs in 10–20%.

#### 1.2.2. Necrotizing Enterocolitis (NEC)

NEC is a serious gastrointestinal disorder in neonates characterized by inflammation, ischemia, and infection, which can lead to bowel necrosis and, in severe cases, perforation. It is the most common surgical emergency in neonates. Its occurrence is reported as 1–3 per 1000 live births worldwide, and it has been documented to affect 2–9% of very-low-birth-weight infants, with a mortality rate ranging from 20% to 50% [10].

#### 1.2.3. Postoperative and Hospital-Acquired IAI

Healthcare-associated IAIs, including post-surgical abscesses, tertiary peritonitis, and anastomotic leaks, form a smaller but clinically important subset, especially in neonatal and oncologic-surgery patients.

### 1.3. Bacterial Species Implicated in Pediatric IAI Etiology

IAIs in children are primarily caused by multiple microbes that reflect the natural microorganisms in the gastrointestinal tract. Common bacterial pathogens involved in IAIs include the body’s own gut flora, including Enterobacteriaceae (*E. coli* and *Klebsiella* spp.), Viridans group *Streptococcus*, and anaerobic bacteria such as *Bacteroides* species [11].

In children with perforated appendicitis, the most isolated microorganisms were *E. coli* (80.14%) and *P. aeruginosa* (7.45%). Furthermore, 5.31% of the *E. coli* isolates were identified as ESBL-producing strains [12].

In a multisite London study of pediatric appendectomies, the most common isolates were *E. coli* (42.0%), *P. aeruginosa* (21.0%), Milleri *Streptococcus* spp. (14.3%), and *B. fragilis* (5.9%). Polymicrobial infection was common, occurring in 32 out of 73 cases [13].

In a large retrospective cohort of children with appendicitis, Gram-negative rods accounted for 67.6%, Gram-positive cocci for 21.5%, and anaerobes for 10.9% of isolates. Milleri-group streptococci were the most frequently isolated Gram-positive organisms (44.7% of Gram-positives), and patients with Gram-positive cocci had longer hospital stays and more complicated diseases [14].

The main aerobic isolates in pediatric IAI are *E. coli* and *Enterococcus* spp.; the primary anaerobic bacteria include hte *B. fragilis* group, *Peptostreptococcus* spp., and *Clostridium* spp [15].

For NEC specifically, a polymicrobial pattern is common, involving enteric Gram-negative rods (*E. coli*, *Klebsiella*, and *Enterobacter*), Gram-positive organisms (*Staphylococcus* and *Enterococcus* spp.), and anaerobes, which play varying roles [16,17].

### 1.4. Collected Specimens

Microbiological evaluation is essential for guiding therapy. The following specimens are collected:

Peritoneal fluid is the most important specimen, aspirated during surgery from the abscess cavity or peritoneal lavage. Two types of samples are usually sent for microbiological analysis: either an intraoperative swab wiped along the serosa of the appendix or intra-abdominal fluid aspirated and sent directly to microbiology.

Intraoperative swabs from the appendix serosa, peritoneum, or abscess wall can be analyzed in a clinical laboratory.

Blood cultures are rarely positive in cIAI (<10%), and thus are mostly unreliable, but they remain important for patients with systemic sepsis or immunocompromised conditions.

Tissue specimens from the resected appendix or bowel (in NEC) are sent for both histopathology and culture.

### 1.5. Surgical Procedures

The surgical approach varies by diagnosis and severity:

Laparoscopic appendectomy is performed in most cases of appendicitis (96.55% in one large series), with conversion to open surgery necessary in about 3.88%. It is now the standard of care in pediatric centers. In cases of perforation with a localized abscess, initial non-operative management (antibiotics ± percutaneous drainage) followed by interval appendectomy at 6–8 weeks is an accepted option. Diffuse peritonitis resulting from perforated appendicitis usually requires urgent appendectomy with peritoneal washout [12].

Nonsurgical support suffices in about 50–75% of NEC cases. Surgical management involves intestinal resection and ostomy creation, most often in the terminal ileum, and the timing of surgery is crucial for improving survival. Primary peritoneal drainage (PPD) is sometimes used either as a temporary measure or as a definitive treatment in very premature or unstable neonates.

Image-guided percutaneous drainage (using ultrasound or CT guidance) is preferred for accessible, clearly defined collections of IA abscesses. Surgical drainage becomes necessary when percutaneous access is not possible, when intestinal source control is needed, or if the patient does not improve with drainage and antibiotics.

Other IAIs (volvulus, intussusception, incarcerated hernia, and Meckel’s diverticulum with perforation) require urgent surgical intervention, including peritoneal lavage and primary repair or resection, depending on viability.

### 1.6. Antibiotic Therapy

Initial therapy is empirical, since microbiological results take 24–72 h. IAIs caused by susceptible bacteria can be managed with intravenous (IV) beta-lactam/beta-lactamase inhibitor combinations (e.g., ticarcillin–clavulanic acid or piperacillin–tazobactam), or a non-pseudomonal carbapenem (e.g., ertapenem) [11]. Felber et al. reported that MDR resistance (to piperacillin–tazobactam and imipenem) was correlated with the cIAI severity (perforated appendicitis) [18].

#### 1.6.1. Low-Risk Pediatric cIAI

Acceptable broad-spectrum antimicrobial regimens for pediatric patients with cIAI include an aminoglycoside-based regimen, a carbapenem (imipenem, meropenem, or ertapenem), a β-lactam/β-lactamase inhibitor combination (piperacillin–tazobactam or ticarcillin–clavulanate), or an advanced-generation cephalosporin (cefotaxime, ceftriaxone, ceftazidime, or cefepime) with metronidazole [19].

#### 1.6.2. Higher-Risk Pediatric cIAI

Piperacillin–tazobactam, imipenem–cilastatin, or meropenem are preferred for empiric therapy in higher-risk pediatric patients older than 1 month with community-acquired or hospital-acquired IAI. Ceftazidime plus metronidazole is an alternative for patients with severe β-lactam reactions; aztreonam, metronidazole, and vancomycin may also be considered [20].

#### 1.6.3. Perforated Appendicitis

The Surgical Infection Society (SIS) recommends piperacillin–tazobactam or ertapenem for perforated appendicitis. The IMPACT study found that piperacillin–tazobactam had significantly lower rates of postoperative intra-abdominal abscesses (6% vs. 24%) than ceftriaxone/metronidazole [21].

#### 1.6.4. Preterm Neonates

Antibiotic regimens recommended for preterm infants with intra-abdominal infections, primarily necrotic enterocolitis (NEC), are ampicillin and gentamicin with either metronidazole or clindamycin, or piperacillin–tazobactam with gentamicin. Metronidazole is the anti-anaerobic agent of choice for combination therapy [21].

#### 1.6.5. Anti-Anaerobic Coverage

Metronidazole is recommended as the preferred anti-anaerobic agent in combination regimens for empiric therapy of low- and high-risk patients (Grade 1-A) [22].

Antibiotic susceptibility testing showed that tazobactam/piperacillin and meropenem inhibited the growth of 96.9–100% of the main bacteria identified. *E. coli* and *P. aeruginosa* were more susceptible to amikacin than gentamicin, supporting tazobactam/piperacillin or meropenem as reasonable first-line options [20,23].

A 5-year retrospective study involving 333 children at St. Louis Children’s Hospital directly related to this age group shows that patients initially treated with broad-spectrum antibiotics (meropenem, piperacillin–tazobactam, fourth-generation cephalosporins) had significantly higher rates of post-appendectomy abscesses (PAA, 25% versus 12%, *p* < 0.01) and longer hospital stays (LOS, 7.0 versus 5.7 days, *p* < 0.01) compared to those who received ceftriaxone plus metronidazole. Discharging patients on antibiotics beyond infection resolution was also associated with higher PAAs (22% versus 9%, *p* < 0.01). The broader-spectrum therapy, which correlated with worse outcomes, can be explained by microbiome disruption, the development of resistant organisms, and, possibly, an altered immune response [24,25]. Therefore, according to the SIS 2026 guidelines, the duration of antibiotic administration after source control should be limited to 5 days for standard cIAI in pediatric patients [21]. For post-appendectomy abscesses (PAAs), the duration should not exceed 7 days. Early oral step-down to high-bioavailability agents such as amoxicillin–clavulanic acid, ciprofloxacin, sulfamethoxazole–trimethoprim, or cephalexin with metronidazole is appropriate once the fever has subsided and the patient can tolerate oral intake. Other studies investigated the use of moxifloxacin [26], amoxicillin–clavulanic acid [27], ceftazidime–avibactam [28] and colistin [29] as potential alternatives to those indicated by the SIS guidelines [21].

However, there is limited pediatric-specific data on the microbiological profiles and antimicrobial susceptibility of intra-abdominal infections (IAIs). This gap is important because it results in reliance on management protocols based on adult guidelines, which may not fully apply to children. Therefore, the present study aims to carefully examine the causes of IAIs requiring surgical intervention in children aged 0 to 16 years at a tertiary referral center and to analyze the susceptibility patterns of Gram-positive and Gram-negative bacterial pathogens. Additionally, we investigated how IAI type, patient age, detected pathogens, and their antimicrobial susceptibility are related. By providing a detailed analysis of these relationships, our findings could help improve the accuracy and effectiveness of treatment strategies for children with intra-abdominal infections.

## 2. Results

### 2.1. Baseline Characteristics of the Pediatric Patients

A total of 177 pediatric patients were included in the study, of whom 64 (36.16%) were girls, and 113 (63.84%) were boys (Table 1). The mean age (years) was 8.73 ± 4.63, without significant differences between girls and boys (8.66 ± 4.73 vs. 8.77 ± 4.59, *p* = 0.98). Boys’ dominance was seen across all age groups. The largest percentage of patients was in the 11–16-year age group (44.07%), followed by those aged 7–10 years (22.03%). Age distribution varied between boys and girls, but the correlation between age groups and gender was not statistically significant (*p* = 0.29, Table 1).

Most IAIs were primary infections (170/177, 96.05%). Only 3.95% (7/177) were SSIs. Appendicitis was the most frequent diagnosis (91/177, 53.53%), followed by IA abscesses (56/177, 32.94%) and peritonitis (8/177, 4.71%). Severe cases of appendicitis were numerous: perforated, with generalized peritonitis (43/177, 25.29%) and gangrenous (23/177, 13.53%). Gangrenous appendicitis and appendicitis with generalized peritonitis were susceptible to generating SSIs in pediatric patients (28.57% and 42.86%, respectively) (Table 1).

### 2.2. Distribution of Isolated Bacterial Species

Because intra-abdominal infections frequently originate from the complex gastrointestinal microbiota, bacterial isolates were analyzed both as single pathogens and as polymicrobial combinations. The analysis of polymicrobial patterns was performed based on Gram-staining characteristics and the number of bacterial species isolated, as described in Section 2.3. Only microorganisms that fulfilled the predefined criteria for etiological significance (i.e., isolation from clinically relevant intra-abdominal specimens and quantitative culture results consistent with infection) were included in the analysis. This approach is consistent with current clinical microbiology practice and with published guidelines that describe the polymicrobial nature of intra-abdominal infections [19,24,25].

Among IAIs caused solely by Gram-negative bacteria (1GN), *E. coli* was the most frequently isolated pathogen (83.33%), followed by *Klebsiella* spp. and *P. aeruginosa*, each identified in 6.41% of cases (Table 2). *Enterobacter* spp. and *C. freundii* were rarely identified (2.56% and 1.28%, respectively).

In the 1GP group, *S. aureus* was the dominant microorganism (60%). In comparison, various *Enterococcus* spp. and *Streptococcus* spp. were also detected in 23.64% and 14.55% of pediatric patients, respectively, and accounted for the remaining isolates. *Gemella morbillum* was identified in a single case (1.82%, Table 2).

Bacterial coinfections were characterized based on the combinations of Gram-negative and Gram-positive pathogens identified in the same clinical specimen.

Infections involving two Gram-negative bacteria (2GN) most often included pairs of *E. coli* and *P. aeruginosa* (52.94%) or *Klebsiella* spp. (17.65%), or *Acinetobacter baumannii* (11.76%, Table 3). Two GN species were observed only in polymicrobial IAIs: *A. baumannii* (2/177) and *Hafnia alvei* (1/177; Table 2). These patterns reflect the typical microbial etiology of intra-abdominal infections, which often involve enteric Gram-negative bacteria along with Gram-positive cocci from the gastrointestinal microbiota.

The most frequently identified 1GN+1GP associations involved *E. coli* in combination with *Streptococcus* spp. (45.83%), or *Enterococcus* spp. (20.83%), or *S. aureus* (8.33%, Table 2). Rare associations included *C. freundii* + *Enterococcus* spp., *H. alvei* + *Streptococcus* spp., and *Klebsiella* spp. + *Streptococcus* spp. or *Enterococcus* spp., *Enterobacter* spp., and *P. aeruginosa* + *Enterococcus* spp. Each of them had 4.17% (Table 2).

Triple bacterial associations were rarely identified in the analyzed cohort. They most frequently include enteric Gram-negative bacteria (e.g., *E. coli* and *P. aeruginosa*) and Gram-positive cocci, such as *Streptococcus* spp. or *Enterococcus* spp. (Table 2). These findings are consistent with previously reported microbiological patterns of complicated intra-abdominal infections, in which mixed aerobic flora may be isolated from clinical specimens obtained directly from the site of infection.

In almost all cases of SSI, double and triple bacterial associations were identified (2GN and 2GN+1GP). Only in one SSI, after inguinal lymph node excision, were 1GP bacteria identified (Table 2).

**Figure 1:**
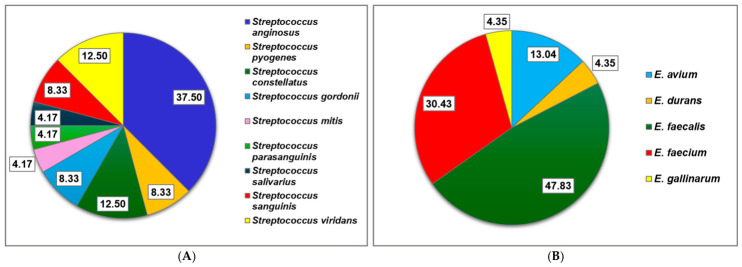

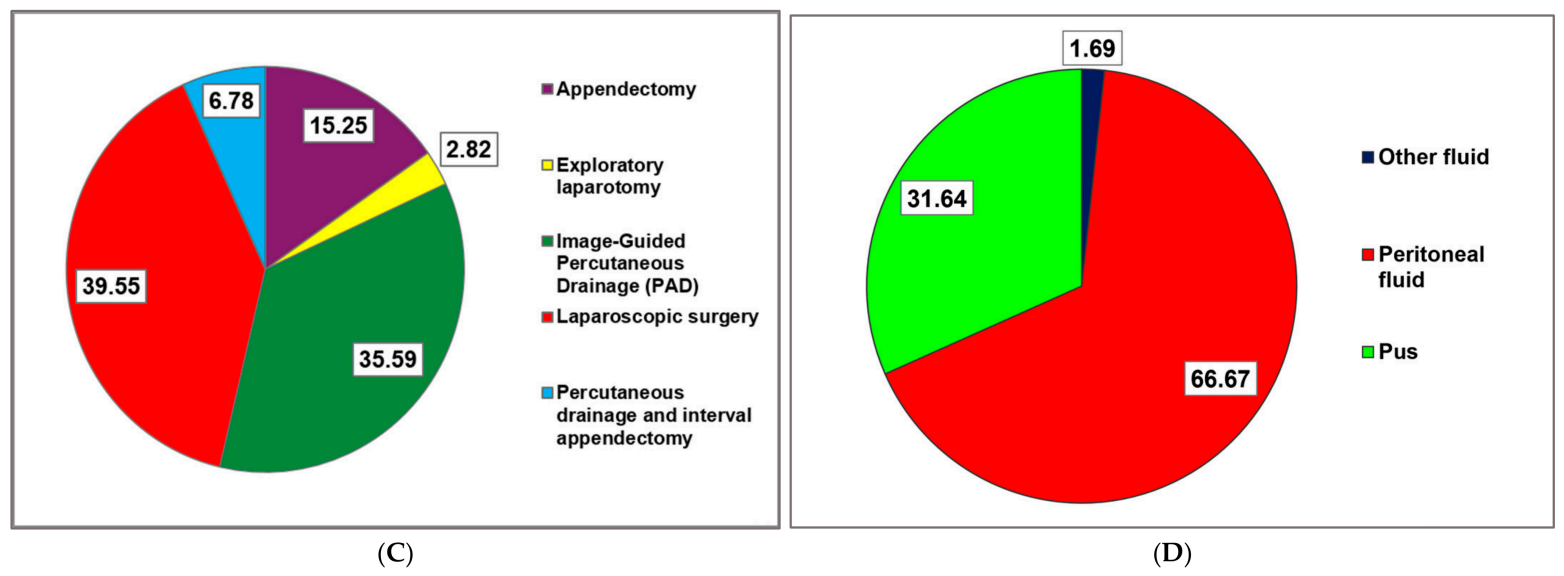
*Streptococcus* (**A**) and *Enterococcus* (**B**) identified species. Surgical interventions (**C**) and biological specimens (**D**).

Nine species of *Streptococcus* (Figure 1A) and 5 species of *Enterococcus* (Figure 1B) were identified. The most common were *Streptococcus anginosus* (37.50%) and *E. faecalis* (47.83%). *Enterobacter cloacae* were the only *Enterobacter* spp. representative.

The most used surgical techniques were laparoscopy (39.55%) and image-guided percutaneous drainage (35.59%; Figure 1C), while the most collected biological specimens were peritoneal fluid (66.67%) and pus (31.64%; Figure 1D).

### 2.3. Antimicrobial Susceptibility Patterns

Susceptibility percentages were calculated as the proportion of susceptible isolates tested among the total number of identified strains.

#### 2.3.1. Gram-Negative Bacteria

*E. coli* was expected to have susceptibility to fourth-generation cephalosporins (94.54%), followed by amoxicillin-clavulanic acid (89.47%), monobactams (95.83%), carbapenems (91.66–100%), aminoglycosides (84.62%), ceftriaxone (66.67%), fluoroquinolones (66.67–73.33%), piperacillin–tazobactam (90%), tigecycline, fosfomycin and colistin (100%) (Table 3). Notable resistance to ampicillin, ampicillin–sulbactam, piperacillin, sulfamethoxazole–trimethoprim, tetracycline, and 2nd-generation cephalosporins (91–100%) was also anticipated (Table 3).

**Table 3:** The main Gram-negative bacteria susceptibility patterns to various antibiotic drugs.

Antibiotic	*n*				Expected (%)	
	Total Tested	Susceptible	Resistant	Not Tested	Susceptibility	Resistance
***E. coli* (*n* = 65)**						
Ampicillin	37	3	34	28	8.10	**91.89**
Ampicillin–Sulbactam	5	0	5	60	0.00	**100.00**
Amoxicillin–Clavulanic acid	57	51	6	8	**89.47**	10.53
Piperacillin	11	0	11	54	0.00	**100.00**
Piperacillin–Tazobactam	10	9	1	55	**90.00**	10.00
Monobactams	48	46	2	17	**95.83**	4.17
Aminoglycosides	26	22	4	39	**84.62**	15.38
Colistin	1	1	0	64	**100.00**	0.00
Fosfomycin	2	2	0	63	**100.00**	0.00
Tetracycline	15	0	15	50	0.00	**100.00**
Sulfamethoxazole–Trimetoprim	25	2	23	40	8.00	**92.00**
Tigecycline	2	2	0	63	**100.00**	0.00
1st-gen. Cephalosporins	12	5	7	53	41.66	**58.34**
2nd-gen. Cephalosporins	3	0	3	62	0.00	**100.00**
4th-gen. Cephalosporins	55	52	3	10	**94.54**	5.46
3rd-gen. Cephalosporins						
Ceftazidime	6	3	3	59	50.00	50.00
Ceftriaxone	18	12	6	47	**66.67**	33.33
Carbapenems						
Meropenem	12	11	1	53	**91.66**	8.33
Ertapenem	15	15	0	50	**100.00**	0.00
Imipenem	12	12	0	53	**100.00**	0.00
Fluoroquinolones						
Ciprofloxacin	15	11	4	50	**73.33**	26.67
Levofloxacin	12	8	4	53	**66.67**	33.33
***Klebsiella* spp. (*n* = 5)**						
Ampicillin	4	0	4	1	0.00	**100.00**
Amoxicillin–Clavulanic acid	4	3	1	1	**75.00**	25.00
Piperacillin	1	0	1	4	0.00	**100.00**
Piperacillin–Tazobactam	1	1	0	4	**100.00**	0.00
Monobactams	2	1	1	3	50.00	50.00
Aminoglycosides	4	3	1	1	**75.00**	25.00
Sulfamethoxazole–Trimetoprim	2	1	1	3	50.00	50.00
1st-gen. Cephalosporins	2	2	0	3	**100.00**	0.00
4th-gen. Cephalosporins	3	3	0	2	**100.00**	0.00
3rd-gen. Cephalosporins						
Ceftazidime	1	1	0	4	**100.00**	0.00
Ceftriaxone	2	1	1	3	50.00	50.00
Carbapenems						
Meropenem	1	1	0	4	**100.00**	0.00
Ertapenem	2	2	0	3	**100.00**	0.00
Fluoroquinolones						
Ciprofloxacin	2	1	1	3	50.00	50.00
Levofloxacin	1	1	0	3	**100.00**	0.00
***P. aeruginosa* (*n* = 5)**						
Aminoglycosides	5	5	0	0	**100.00**	0.00
Colistin	4	4	0	1	**100.00**	0.00
Piperacillin	1	0	1	4	0.00	**100.00**
Piperacillin–Tazobactam	1	1	0	4	**100.00**	0.00
4th-gen. Cephalosporins	2	1	1	3	50.00	50.00
3rd-gen. Cephalosporins						
Ceftazidime	1	1	0	4	**100.00**	0.00
Ceftriaxone	1	1	0	4	**100.00**	0.00
Carbapenems						
Meropenem	2	2	0	3	**100.00**	0.00
Imipenem	1	1	0	4	**100.00**	0.00

Data is expressed as numbers and percentages: *n*—number (frequency); %—percentage (relative frequency); 1st-, 2nd-, 3rd-, 4th-gen. represent the generation of cephalosporins. Cephalosporins of the fourth generation usually refer to Cefepime. Monobactams usually refer to Aztreonam. Aminoglycosides usually refer to Amikacin. Macrolides usually refer to Azithromycin. Bolded values from the Susceptibility and Resistance columns are >50%.

Amoxicillin-clavulanic acid, aminoglycosides, carbapenems, piperacillin-tazobactam, 1st- and 4th-generation cephalosporins, ceftazidime, and levofloxacin were expected to be active against *Klebsiella* spp. (75–100%) (Table 3). The notable resistance of *Klebsiella* spp. to ampicillin and piperacillin (100%) was also anticipated (Table 3).

*P. aeruginosa* was expected to be susceptible to aminoglycosides, colistin, 3rd-generation cephalosporins, piperacillin-tazobactam, and carbapenems (100%) and resistant to piperacillin (Table 3).

*Enterobacter* spp. (*Enterobacter cloacae*). and *C. freundii* were expected to be susceptible to ertapenem and 4th-generation cephalosporins (Table 4).

#### 2.3.2. Gram-Positive Bacteria

Aminoglycosides, fluoroquinolones, vancomycin, linezolid, teicoplanin, and tigecycline were expected to be effective against *S. aureus* and *Enterococcus* spp. (57.14–69.23%) (Table 5). Resistance to ampicillin and amoxicillin–clavulanic acid was anticipated for both GP bacteria (57.14–100%) (Table 5).

*S. aureus* was also expected to be susceptible to clindamycin, fusidic acid, fosfomycin, 4th-generation cephalosporins, ceftriaxone, trimethoprim-sulfamethoxazole, and moxifloxacin (57.14–100%). Moreover, carbapenems, piperacillin-tazobactam, and tetracycline had an expected efficacy against half of the *S. aureus* strains (Table 5). Penicillin, oxacillin, ampicillin–sulbactam, 1st- and 2nd-generation cephalosporins, macrolides, and ceftazidime were expected to have no inhibitory effects against *S. aureus* (Table 5).

*Enterococcus* spp. strains’ susceptibility to ampicillin–sulbactam and macrolides (100%) and resistance to tetracycline (100%) were also anticipated (Table 5).

*Streptococcus* spp. was expected to be susceptible to almost all tested antibacterial drugs (ceftriaxone, macrolides, chloramphenicol, teicoplanin, clindamycin, and penicillin), and resistant to ceftazidime (Table 5).

Vancomycin was anticipated to be effective against *Gemella morbillorum*.

### 2.4. The Incidence of IAIs/SSIs and Pathogen Types in Pediatric Patients of Different Age Groups

Pediatric patients’ IAI types and pathogen types are displayed for each age group in Table 6 and Table 7.

The most severe IAIs were polymicrobial (Table 7), and empiric antibiotherapy had an essential role in clinical outcomes.

A total of 75% of infants (<1 year) had IA abscesses; the others had other very severe IAIs (Table 6). In 91% of collected specimens of neonates, 1GN (50%) and 1GP (41.67%) were identified; 2GN was detected in only 8.33% of cases (Table 7).

Children aged 1–3 years had mainly IA abscesses (38.10%) and peritonitis (19.05%); three pediatric patients had SSIs (14.28%) (Table 6). In most of the collected specimens, 1GN (47.62%) and 1GP (33.33%) were identified. Only in 19% of cases were dual bacterial associations detected (1GN+1GP and 2GN) (Table 7).

Children aged 4–6 years had, in equal percentage (25.93%), IA abscesses and appendicitis (perforated, with generalized peritonitis), followed by another severe form of appendicitis (perforated, with localized peritonitis, 14.81%) (Table 6). In 29.63% of specimens collected, double and triple GN and GP bacteria associations were detected (Table 7).

Gangrenous appendicitis (30.77%) and another severe form (perforated, with generalized peritonitis, 28.21%) had the highest incidence in children aged 7–10 years (Table 6). A total of 12.82% had IA abscesses; moreover, two SSIs occurred in this age group (Table 6). In over 40% of cases, double bacterial associations were identified (Table 7).

IA abscesses (34.62%), and the most severe form of appendicitis (perforated, with generalized peritonitis, 32.05%) were commonly diagnosed in 11–16-year-old children. Two SSIs occurred in this age group (Table 6). In almost 20% of cases, GN and GP bacterial associations were involved (Table 7).

### 2.5. Empirical Antibiotherapy in Polimicrobial IAIs/SSIs

#### 2.5.1. Double Gram-Negative Bacteria Associations (2GN)

Of 17 IAIs with 2GN bacteria, over 50% involved a dual association between *E. coli* and *P. aeruginosa* (Table 8).

Fourth-generation cephalosporins, monobactams, and aminoglycosides could be the main choices for IAIs caused by two associated GN bacteria (Table 8).

Fourth-generation cephalosporins, monobactams, and ciprofloxacin have been reported to have potential efficacy for IAIs caused by *C. freundii* + *Enterobacter* spp.

Multiple choices could be available in IAIs caused by *E. coli* + *A. baumannii and Enterobacter* spp. + *Klebsiella* spp. (Table 8).

Amoxicillin–clavulanic acid could also be effective for IAIs caused by *E. coli* associated with *Klebsiella* spp. and *P. aeruginosa* (Table 8).

#### 2.5.2. Dual Bacterial Associations 1GN+1GP in IAIs

Fourth-generation cephalosporins could be effective in almost all combinations (7/9), followed by monobactams (6/9), amoxicillin–clavulanic acid (5/9), and carbapenems (5/9) (Table 9).

Our results suggest that aminoglycosides, ceftriaxone, moxifloxacin, fusidic acid, linezolid, tigecycline, tetracycline, and trimethoprim–sulfamethoxazole could be used in IAIs with *E. coli* + *S. aureus* (Table 9). Linezolid and colistin showed potential benefits in IAIs caused by *P. aeruginosa* + *Enterococcus* spp. (Table 9).

#### 2.5.3. Triple Bacterial Associations (1GN+2GP and 2GN+1GP)

For both triple bacterial associations (*E. coli* + *P. aeruginosa* + *Streptococcus* spp. and *E. coli* + *Enterococcus* spp. + *Streptococcus* spp.), the empiric therapy could be based on 4th-generation cephalosporins, aminoglycosides, amoxicillin–clavulanic acid, and monobactams.

## 3. Discussion

### 3.1. Principal Findings

#### 3.1.1. IAI Type

Most IAIs were primary infections, whereas SSIs accounted for <4% of pediatric cases. Appendicitis was the most frequent diagnosis, followed by IA abscesses and peritonitis. Severe cases of appendicitis were numerous: perforated, with generalized peritonitis, and gangrenous. Gangrenous appendicitis and appendicitis with generalized peritonitis were susceptible to generating SSIs in pediatric patients.

#### 3.1.2. GN and GP Pathogens

Among IAIs caused solely by Gram-negative bacteria, *E. coli* was the most frequently isolated pathogen (83.33%), followed by *Klebsiella* spp. and *P. aeruginosa*, each identified in 6.41% of cases. Our findings align with the literature data, which report that these three GN bacteria are the most common pathogens involved in IAIs [30]. *Enterobacter* spp. was less frequent (2.56%).

In collected specimens, *S. aureus* was the dominant Gram-positive bacterium (60%), followed by *Enterococcus* spp. and *Streptococcus* spp. (in 23.64% and 14.55% of pediatric patients). In their recent study, Banerjee et al. reported that *Enterococcus* spp. was the most common pathogen in perforation peritonitis, and *S. aureus* was the most common pathogen in IA abscesses [31]. *Streptococcus* spp. was involved in primary and secondary peritonitis and intra-abdominal abscesses [32,33].

Moreover, *C. freundii* (2 cases) and *Gemella morbillorum* (a single case) were identified.

Our findings align with those in the literature: in a study of complicated pediatric appendicitis, the most common bacteria were *E. coli* (66.7%), α-*Streptococcus* (46.3%), *P. aeruginosa* (18.5%), *E. avium* (16.7%), γ-*Streptococcus* (16.7%), and *K. oxytoca* (11.1%) [19]. Furthermore, in their recent retrospective study, Felber et al. identified all species found in our study, with varying frequencies [20]. *E. coli* had the highest incidence (312 cases), followed by *S. aureus* (27), *P. aeruginosa* (75), and *Streptococcus* spp., including *S. anginosus* (66), *S. constellatus* (38), *S. gordonii* (2), *S. salivarius* (3), *S. sanguinis* (3), *S. parasanguinis* (3), and *S. pyogenes* (4). *Enterococcus* spp. included *E. avium* (28), *E. durans* (2), *E. faecalis* (12), *E. faecium* (7), and *E. gallinarum* (2). Other bacteria observed included *Enterobacter cloacae* (2), *Gemella morbillorum* (7), *C. freundii* (10), and *Klebsiella* spp., including *K. oxytoca* (16) and *K. pneumoniae* (14).

#### 3.1.3. GN and GP Bacteria Associations

Infections involving two Gram-negative bacteria most often involve pairs of *E. coli* and *P. aeruginosa*, *Klebsiella* spp., or *Acinetobacter baumannii*. Two GN species were identified only in polymicrobial IAIs: *A. baumannii* and *Hafnia alvei*. These patterns reflect the typical microbial etiology of intra-abdominal infections, which often involve enteric Gram-negative bacteria, along with Gram-positive cocci from the gastrointestinal microbiota [34].

The most frequently identified 1GN+1GP associations involved *E. coli* in combination with *Streptococcus* spp., or *Enterococcus* spp., or *S. aureus*. Rare associations included *C. freundii* + *Enterococcus* spp., *H. alvei* + *Streptococcus* spp., and *Klebsiella* spp. + *Streptococcus* spp. or *Enterococcus* spp., *Enterobacter* spp., and *P. aeruginosa* + *Enterococcus* spp.

Regarding coinfections with clinical significance, those involving *Enterococcus* spp. and *Acinetobacter baumannii* had a mortality rate of 62.5%, and those involving *Enterococcus* spp. and *K. pneumoniae* had a mortality rate of 66.7%, underscoring the prognostic significance of specific coinfection combinations [35].

Triple bacterial associations were rarely identified in the analyzed cohort. They included enteric Gram-negative bacteria (e.g., *E. coli* and *P. aeruginosa*) and Gram-positive cocci, such as *Streptococcus* spp. or *Enterococcus* spp.

Our results align with previous studies, which reported that, in peritoneal-fluid cultures of complicated pediatric appendicitis, the major identified bacteria included *E. coli* (66.7%), α-*Streptococcus* (46.3%), *P. aeruginosa* (18.5%), *Enterococcus avium* (16.7%), γ-*Streptococcus* (16.7%), and *K. oxytoca* (11.1%). They evidenced the breadth of polymicrobial coinfection patterns [23].

Polymicrobial etiology was linked to a fourfold increased risk of complicated appendicitis. Gram-negative bacteria and polymicrobial cultures in both the peritoneal fluid and the appendiceal lumen were independently associated with higher rates of complicated appendicitis [36].

### 3.2. Antibacterial Susceptibility Patterns

#### 3.2.1. GN Bacteria

Our findings revealed that piperacillin–tazobactam and carbapenems were expected to be effective against almost all GN pathogens identified in surgical specimens of pediatric patients.

Fourth-generation cephalosporins, amoxicillin–clavulanic acid, fluoroquinolones, and aminoglycosides were anticipated to be effective against Enterobacteriaceae (*E. coli* and *Klebsiella* spp.).

*E. coli* was also expected to have susceptibility to monobactams, ceftriaxone, tigecycline, fosfomycin, and colistin. First-generation cephalosporins and ceftazidime were expected to be active against *Klebsiella* spp.

*P. aeruginosa* was anticipated to be susceptible to colistin and 3rd-generation cephalosporins.

On the other hand, the main GN bacteria were expected to be resistant to piperacillin, while the Enterobacteriaceae strains’ resistance to ampicillin was also expected. Moreover, *E. coli*’s notable resistance to ampicillin–sulbactam, sulfamethoxazole–trimethoprim, tetracycline, and 2nd-generation cephalosporins was also anticipated. These resistance patterns align with the literature, which indicates that ESBL-producing pathogens are an emerging concern in pediatric coinfections. A significant proportion of peritoneal cultures in children with perforated appendicitis yielded ESBL-positive *E. coli* (57.9% of all *E. coli* isolates in one cohort), suggesting bowel colonization with resistant bacteria even in the community setting [13].

#### 3.2.2. GP Bacteria

Aminoglycosides, fluoroquinolones, vancomycin, linezolid, teicoplanin, and tigecycline were expected to be effective against *S. aureus* and *Enterococcus* spp. Vancomycin was also anticipated to be effective against *Gemella morbillorum*.

*S. aureus* was also expected to be susceptible to clindamycin, fusidic acid, fosfomycin, 4th-gen. cephalosporins, ceftriaxone, trimethoprim–sulfamethoxazole, and moxifloxacin (57.14–100%). Moreover, carbapenems, piperacillin–tazobactam, and tetracycline had an expected efficacy against half of the *S. aureus* strains tested.

*Enterococcus* spp. strains’ susceptibility to ampicillin–sulbactam and macrolides (100%), and *Streptococcus* spp. strains’ susceptibility to ceftriaxone, macrolides, chloramphenicol, teicoplanin, clindamycin, and penicillin were also anticipated.

On the other hand, resistance to ampicillin and amoxicillin–clavulanic acid was expected for both *Enterococcus* spp. and *S. aureus*. Penicillin, oxacillin, ampicillin–sulbactam, 1st-and 2nd-generation cephalosporins, macrolides, and ceftazidime were expected to have no efficacy against *S. aureus*. *Enterococcus* spp. strains’ resistance to tetracycline and *Streptococcus* spp. strains’ resistance to ceftazidime were also anticipated.

### 3.3. Empirical Antibiotherapy in Pediatric IAIs

The microbiology of complicated IAI is predominantly determined by the site of GI-tract integrity loss and the degree of disruption. Because the lower GI tract harbors hundreds of bacterial species at high concentrations, cIAI arising from the lower GI tract is likely polymicrobial [37]. Infections originating in the colon are associated with facultative and obligate anaerobic bacteria and Gram-negative pathogens [34].

The polymicrobial and co-infective nature of pediatric IAIs has direct treatment implications. Empirical regimens must cover both aerobic Gram-negative bacteria (mainly *E. coli*, *Klebsiella* spp., and *P. aeruginosa*) and obligate anaerobes (*B. fragilis* group).

Where *P. aeruginosa* was identified, empirical therapy required extending anti-pseudomonal beta-lactam coverage [14]. Isolation of *Pseudomonas* spp. from intra-operative sampling was associated with a greater length of hospital stay (7.0 vs. 5.0 days; *p* = 0.011). When *P. aeruginosa* co-infects with *E. coli*, empirical regimens using amoxicillin–clavulanic acid or first-generation cephalosporins are inadequate, as they have no activity against *Pseudomonas* [14,34]. Antibiotic susceptibility testing shows that piperacillin–tazobactam and meropenem inhibited 96.9–100% of the main bacteria identified across all co-infecting species, making these drugs the best choices when polymicrobial infection is suspected [23]. Our findings regarding susceptibility patterns confirm these aspects: piperacillin–tazobactam and carbapenems were expected to be effective against almost all GN pathogens identified in surgical specimens from pediatric patients and against 50% of *S. aureus* strains. The *Surgical Infection Society 2025 guidelines* specifically address coinfection burden: piperacillin–tazobactam or ertapenem are recommended for perforated appendicitis, because piperacillin–tazobactam shows significantly lower rates of postoperative intra-abdominal abscesses (6% vs. 24%) compared to ceftriaxone/metronidazole—a difference partly due to broader polymicrobial coverage, including anti-pseudomonal activity [21].

The present study suggests that fourth-generation cephalosporins and aminoglycosides may be effective against all double- and triple-GN and GP bacterial associations involved in pediatric IAIs.

Monobactams, amoxicillin–clavulanic acid, and carbapenems had expected benefits in 2GN IAIs, while ceftriaxone, moxifloxacin, fusidic acid, linezolid, tigecycline, tetracycline, and trimethoprim–sulfamethoxazole could be used in IAIs with *E. coli* + *S. aureus*; linezolid and colistin could have potential benefits in IAIs caused by *P. aeruginosa* + *Enterococcus* spp.

### 3.4. Empirical Antibiotherapy in IAIs of Pediatric Patients of Different Age Groups

We classified pediatric patients into these age groups (<1 year, 1–3 years, 4–6 years, 7–10 years, and 11–16 years), because each category has distinct characteristics.

Entirely different entities dominate the neonatal and early-infant period. Necrotizing enterocolitis (NEC) is the most severe gastrointestinal emergency in preterm neonates, estimated to affect approximately 6% of very-low-birthweight infants (<1500 g), with mortality reaching 50.9% in extremely-low-birthweight infants with surgical disease [13]. In perforated appendicitis, the average number of different species per specimen rises to 4.54, compared to 2.88–3.3 in earlier disease stages. The perforation rate in preschool children is inversely proportional to patient age, occurring in 100% of those aged under 1 year, 91% aged 1–2 years, 76% aged 2–3 years, 73% aged 3–4 years, and 57% aged 4–5 years. The risk of perforation increased proportionately with the duration of symptoms [38]. By contrast, perforation rates across the broader pediatric age range are reported from 10% to 30%, with children at the extremes of age—particularly those under 2 years—at the highest risk, with rates as high as 90% in that group [39]. Perforation rates are as high as 82% for pediatric patients under 5 years and 100% for those under 1 year [40]. Moreover, the misdiagnosis rate ranges from 28 to 57% in 2-to-12-year-old children and approaches nearly 100% in children younger than 2 years. Delays in diagnosis result from poor communication skills, failure to elicit physical signs in irritable children, atypical presentations, and symptoms that overlap with other disorders. Because of its unusual presentation in children younger than five years old, appendicitis is often misdiagnosed, which leads to increased morbidity.

#### 3.4.1. Empirical Antibiotherapy in Pediatric Patients <1 Year

Clinical presentation varies between infants and preschoolers, though no statistically significant differences were observed in the rate of perforated appendix or postoperative complications between these two youngest groups [24,41]. In all stages, *E. coli* was the dominant species, followed by *Bacteroides* and *Pseudomonas*. For neonatal entities, NEC is associated with fewer anaerobic isolates; aerobic species, including *Klebsiella*, *Enterobacter*, and *Streptococcus* spp., are instead frequently reported [42,43]. Bowel intussusception can generate bowel necrosis in neonates [44,45].

Metronidazole is the anti-anaerobic agent of choice for combination therapy in infants beyond the neonatal period [19]. For lower-risk infants, the recommended treatment is either a combination of ceftriaxone with metronidazole or monotherapy with ertapenem [46]. For pre-term infants with intra-abdominal infections (primarily NEC), the recommended regimens include ampicillin and gentamicin with either metronidazole or clindamycin, or piperacillin–tazobactam with gentamicin [47].

Our findings reveal that *E. coli* and *Enterococcus* spp. were the most frequently identified aerobic pathogens in infant IAIs, and align with these recommendations for empirical therapy in infants with IAIs (RPS = 3.580, *p* < 0.001).

#### 3.4.2. Empirical Antibiotherapy in Pediatric Patients Aged 1–3 Years

IDSA/SIS guidelines base therapeutic recommendations on the severity of infection, which is determined by a combination of patient age, physiological disturbances, and underlying medical conditions. Patients with high risk have a higher chance of treatment failure, including younger age, infections in anatomically unfavorable locations, or healthcare-associated infections [48]. For higher-risk pediatric patients aged 1–3 years, piperacillin–tazobactam and meropenem alone were recommended [49].

The main bacterial isolates detected in collected specimens were *E. coli* and *S. aureus*; our findings align with previous recommendations for this age group (RPS = 3.619, *p* < 0.001).

#### 3.4.3. Empirical Antibiotherapy in Pediatric Patients Aged 4–6 Years

The 4-to-6-year age group marks a significant transitional phase in pediatric intra-abdominal infections (IAIs). It acts as a bridge between two distinct clinical extremes: the very young child (<3 years), who often shows near-universal appendiceal perforation and diffuse peritonitis, due to anatomical immaturity; and the school-age child (>7 years), who typically presents with characteristic symptoms of appendicitis, has perforation rates below 30%, and benefits from the developing omentum’s ability to contain peritoneal contamination by forming a localized abscess [50]. Pediatric patients aged 4–6 years are undergoing a gradual yet incomplete process of anatomical and immunological development, resulting in a mixed clinical picture marked by persistently high perforation rates, some potential for omental containment, and an emerging—though often still absent—classic presentation [39]. We identified double and triple GN and GP associations in almost 30% of the specimens collected.

Intravenous ceftriaxone + metronidazole, ertapenem monotherapy, piperacillin–tazobactam, and meropenem+/− vancomycin were recommended in the empirical protocol [49,51], followed by oral antibiotherapy after discharge [52,53,54].

Most detected pathogens were *S. aureus* and *E. coli* (alone, or in double or triple association with *Streptococcus* spp., *Enterococcus* spp., and *P. aeruginosa*).

Our results align with the therapeutic protocol for children aged 4–6 years (RPS = 2.170, *p* < 0.001).

#### 3.4.4. Empirical Antibiotherapy in Pediatric Patients Aged 7–10 Years

The 7–10-year age group represents the period of first consistent diagnostic clarity in pediatric intra-abdominal infections. The perforation rate remains higher than the adult rate, but has decreased significantly from the severe rates observed in younger age groups. Gangrenous and perforated appendicitis with generalized peritonitis recorded the highest incidence in children aged 7–10 years. Almost all GN and GP pathogens were detected, except *S. aureus*, and, in over 40% of cases, double bacterial associations were identified in collected specimens. The same antibacterial protocol is officially recommended [52,53,54]. Our data also aligns with it (RPS = 1.661, *p* < 0.001).

#### 3.4.5. Empirical Antibiotherapy in Pediatric Patients Aged 11–16 Years

Antimicrobial resistance is a significant concern in clinical settings: ESBL-producing *E. coli* has been found in up to 57.9% of peritoneal isolates from patients with perforated appendicitis in this age group [3], raising questions about the effectiveness of standard empirical treatments [55,56,57]. Furthermore, inflammatory bowel disease (Crohn’s disease) and other non-appendiceal IAI conditions are becoming more common, and their management involves immunosuppressive therapy in ways that are not relevant to younger children [58]. In the present study, IA abscesses in a high percentage (almost 35%), and the most severe forms of appendicitis (perforated, with generalized peritonitis, over 30%) were commonly diagnosed in 11–16-year-old children. GN and GP bacterial associations accounted for almost 20% of cases. SIS-2026 guidelines enriched the therapeutic protocol for pediatric IAIs by including linezolid, based on a possible association with meropenem in septic shock and immunocompromised patients [21]. Our findings align with these data (RPS = 1.192, *p* < 0.001, for almost all known antibacterials included in protocol and RPS = 1.884, *p* < 0.001, for linezolid).

### 3.5. Limitations of the Present Study

#### 3.5.1. The Absence of Anaerobic Pathogen Identification

Antimicrobial susceptibility patterns mainly concern aerobic and facultatively anaerobic bacteria. The current results do not fully define the microbiology of IAIs. Caution is recommended when applying these findings to infections with a significant anaerobic component.

Intra-abdominal infections generally follow a two-phase, synergistic pattern: an initial stage of peritonitis and bacteremia caused by aerobes, followed by a later abscess stage involving anaerobic bacteria [59]. Although *Bacteroides fragilis* is the most well-known pathogen, other anaerobic bacteria, including various species within the *B. fragilis* group, also play a significant role [60]. Anaerobic bacteremia often originates from intra-abdominal sources. This biphasic process explains why initial cultures frequently grow aerobic bacteria like *E. coli*, while anaerobes become more common as abscesses develop over 48–72 h or longer. These bacteria are especially important in perforated appendicitis and abscess formation; neglecting them can lead to underestimating polymicrobial infections [61].

Despite their high actual prevalence, anaerobic isolation rates in pediatric IAI studies vary significantly. This variation mainly results from methodological differences. Anaerobes are often missed in routine microbiology because specialized procedures are required for their transport and growth in environments without oxygen [59].

The frequent detection of anaerobes in pediatric IAI, whether through culture or otherwise, is consistently reflected in antibiotic guidelines. The Infectious Diseases Society of America (IDSA) recommends metronidazole as part of combination therapy for empirical treatment of complicated IAI in children. This inclusion specifically targets intra-abdominal infections caused by anaerobic bacteria, including *Bacteroides*, *Clostridium*, *Eubacterium*, *Peptococcus*, and *Peptostreptococcus* spp. [21,62].

#### 3.5.2. Additional Limitations

○ The absence of molecular identification and in vitro synergy testing for rapid, accurate identification and treatment optimization.
○ The absence of phenotypic methods to detect ESBL-producing pathogens.
○ The absence of identification of fungal pathogens, which are frequently implicated in intra-abdominal infections in immunocompromised pediatric patients [63].
○ The data presented primarily indicate microbiological susceptibility, instead of the clinical efficacy of the regimens.
○ The single-center design and the retrospective methodology may limit the generalizability and preclude causal assessment.

### 3.6. Essential Considerations

Despite the limitations mentioned above, our study provides a comprehensive overview of Gram-positive and Gram-negative pathogens and their antimicrobial susceptibility patterns in pediatric intra-abdominal infections requiring surgical intervention at a tertiary referral center. The diagnosis of IAI was associated with pediatric patients’ age, identified pathogens, and susceptibility patterns. Extensive statistical analysis supports these findings, with detailed correlations across all available data.

The main findings revealed a dominance of Gram-negative bacteria, especially *E. coli*, frequent occurrence of polymicrobial infections in complicated appendicitis and intra-abdominal abscesses, and notable variability in antimicrobial susceptibility among the isolated pathogens.

The prominence of *E. coli* was anticipated, as it constitutes the primary facultative anaerobe in the normal intestinal microbiota and is most commonly involved in perforated appendicitis and secondary peritonitis. Similarly, the high rate of polymicrobial infections reflects bacterial translocation following gastrointestinal perforation or tissue necrosis, in which multiple enteric microorganisms simultaneously contaminate the peritoneal cavity. These microbiological features justify the continued need for broad-spectrum empirical antimicrobial therapy until culture results are available.

Our findings are consistent with previous pediatric studies identifying *E. coli* as the main pathogen in complicated appendicitis, alongside *Klebsiella* spp., *P. aeruginosa*, *Enterococcus* spp., and *Streptococcus* spp. Although the overall bacterial spectrum mirrors that reported in the literature, the susceptibility profiles exhibit local differences, emphasizing the importance of tailoring empirical antibiotic protocols based on institutional microbiological surveillance, rather than solely relying on international guidelines.

Our results also support a stewardship-oriented approach to empirical antimicrobial therapy in pediatric intra-abdominal infections. Ceftriaxone plus metronidazole remains an appropriate empirical regimen for most community-acquired, low-risk infections caused by susceptible Enterobacteriaceae, especially in cases of complicated appendicitis without risk factors for multidrug-resistant pathogens. Piperacillin–tazobactam should be considered for patients with severe or healthcare-associated infections, polymicrobial infections, or when *P. aeruginosa* or resistant Gram-negative bacteria are clinically suspected. Carbapenems should be reserved for microbiologically confirmed infections caused by ESBL-producing or other multidrug-resistant organisms, or for patients with treatment failure or severe sepsis, to preserve their activity and limit antimicrobial resistance. Routine intraoperative peritoneal cultures are essential because they enable early pathogen identification, susceptibility testing, and timely de-escalation from broad-spectrum empirical therapy to targeted treatment. This strategy may reduce unnecessary carbapenem use while maintaining adequate antimicrobial coverage and supporting antimicrobial stewardship programs in pediatric surgical practice.

The originality of our study lies in its effective approach to assessing etiology, IAI type, susceptibility patterns, types of surgical interventions, and age distribution within a pediatric cohort of this size, highlighting an emerging area for further research and understanding.

From an antimicrobial stewardship perspective, our results underscore the importance of obtaining intraoperative microbiological specimens whenever possible. Culture-guided de-escalation enables clinicians to reduce unnecessary exposure to broad-spectrum antibiotics, while ensuring adequate pathogen coverage. This approach can help limit antimicrobial resistance, decrease adverse drug effects, and optimize antibiotic use in pediatric surgical patients.

From a pediatric surgeon’s standpoint, these findings emphasize the fact that managing intra-abdominal infections effectively requires not only appropriate antimicrobial therapy, but also prompt and adequate surgical source control. Understanding local microbiological epidemiology and resistance patterns can guide more effective empirical antibiotic selection during the perioperative period, particularly in cases of perforated appendicitis, diffuse peritonitis, postoperative intra-abdominal infections, or abscess formation. Continued collaboration among pediatric surgeons, infectious-disease specialists, microbiologists, and antimicrobial stewardship teams remains crucial for improving clinical outcomes and preserving antibiotic efficacy.

## 4. Materials and Methods

### 4.1. Study Design and Pediatric Patients

This retrospective observational study was carried out at the “Saint Apostle Andrew” Clinical Hospital in Constanța, including 177 pediatric patients who underwent surgery for intra-abdominal conditions with confirmed intra-abdominal infections from January 2022 to November 2025. The study complied with the ethical standards of the Declaration of Helsinki (Fortaleza revision, 2013) and received approval from the Ethics Committee of “Saint Apostle Andrew” Clinical Hospital, Constanța (approval number: 02/2026).

Pediatric patients’ anonymity was protected by using numeric codes and removing personal identifiers. Intraoperative and postoperative specimens underwent standard microbiological analysis. Infections were classified as monomicrobial or polymicrobial, with antimicrobial susceptibility assessed phenotypically.

### 4.2. Inclusion/Exclusion Criteria

#### 4.2.1. Inclusion Criteria

Pediatric patients of both genders aged 0–16 years were eligible for inclusion if intra-abdominal infection was identified either intra- or postoperatively, and microbiological samples were obtained from the intra-abdominal site.

Pediatric patients undergoing surgery for infectious intra-abdominal conditions, such as complicated appendicitis (perforated appendicitis, appendicular abscess, or generalized peritonitis), intra-abdominal abscess, or diffuse peritonitis, were included in the present study. The term “acute abdomen” refers to the clinical presentation at admission; the definitive diagnosis was established intraoperatively, and confirmed by microbiological findings when intra-abdominal infection was present. Children who developed postoperative surgical site infections (SSIs) were also included; SSIs were diagnosed according to the Centers for Disease Control and Prevention (CDC) criteria as infections involving the intra-abdominal space that occurred within 30 days following surgery, in the absence of implanted material [64].

#### 4.2.2. Exclusion Criteria

Patients aged >16 years.

Cases of uncomplicated acute appendicitis without intra-abdominal infection were excluded.

Patients without microbiological confirmation, those managed non-operatively, or cases with incomplete clinical or laboratory data were excluded.

### 4.3. Data Collection

Demographic data (age, sex), clinical diagnosis, type of surgical intervention, microbiological findings, and antimicrobial susceptibility results were extracted from electronic medical records.

#### 4.3.1. Age and Gender

Pediatric patients were stratified into age groups (<1, 1–3, 4–6, 7–10, and 11–16 years) to evaluate age-related differences in pathogen distribution and antimicrobial susceptibility patterns.

#### 4.3.2. Diagnoses

Diagnoses were classified in primary IAIs (appendicitis, IA abscesses, peritonitis, bowel diseases, and others) and SSIs.

#### 4.3.3. Surgical Interventions

Surgical procedures were categorized as follows: appendectomy, image-guided percutaneous drainage (PAD), percutaneous drainage and interval appendectomy, laparoscopic surgery, and exploratory laparotomy.

### 4.4. Microbiological Analysis

Biological samples (peritoneal fluid, pus, and other intra-abdominal fluids) were collected under sterile conditions during surgical procedures or from postoperative drainage systems. Samples were immediately transported to the microbiology laboratory and processed according to standardized institutional microbiological protocols, to minimize contamination.

Gram-negative and Gram-positive bacteria were identified, and antimicrobial susceptibility testing was performed using the VITEK^®^ 2 automated system (bioMérieux, Marcy-L’Étoile, France) according to the manufacturer’s instructions. Antimicrobial susceptibility results were interpreted according to the European Committee on Antimicrobial Susceptibility Testing (EUCAST) clinical breakpoints (Version 14.0, 2024) [21].

The microbiology department of the clinical laboratory did not perform molecular identification, anaerobic bacteria identification, or in vitro synergy testing.

#### 4.4.1. Results Interpretation

Microorganisms were considered etiologically significant when isolated from clinically relevant specimens obtained directly from the site of infection and in quantities consistent with true infection, rather than contamination. For intra-abdominal fluid samples, the isolation of recognized pathogenic bacteria (Gram-negative or Gram-positive) was considered clinically relevant. Common skin commensals (e.g., coagulase-negative staphylococci) were considered significant only when isolated in pure culture from normally sterile intra-abdominal specimens. When available, quantitative culture thresholds were applied. For peritoneal fluid samples, bacterial growth ≥ 10^3^–10^4^ CFU/mL was considered indicative of clinically significant infection according to institutional microbiology laboratory protocols and commonly accepted microbiological criteria.

Intra-abdominal infections are frequently polymicrobial, due to the complex microbiota of the gastrointestinal tract, involving both aerobic and anaerobic bacteria [19,22]. Therefore, the interpretation of identified bacterial isolates should consider the clinical context, quantitative culture results, and the type of specimen collected.

Satisfactory susceptibility was considered when the percentage of susceptible bacteria was over 50%.

Polymicrobial infections were classified according to the Gram-staining characteristics of the isolated organisms. Dual infections involving one Gram-negative and one Gram-positive bacterium were reported as 1GN+1GP; the order of the abbreviations does not indicate quantitative differences in colony-forming units (CFUs) or relative abundance. Triple associations were similarly categorized (e.g., 2GN+1GP or 1GN+2GP).

#### 4.4.2. Empirical Antibiotherapy

Antimicrobial regimens were evaluated based on susceptibility results for each antimicrobial agent included in commonly used empirical regimens (e.g., β-lactam plus aminoglycoside or β-lactam plus metronidazole) [1]. A combination regimen was considered appropriate when all individual components were reported as susceptible, according to EUCAST criteria.

### 4.5. Follow-Up and SSI Surveillance

Postoperative surveillance for intra-abdominal SSI was conducted during hospitalization and through outpatient follow-up visits or hospital readmissions documented in the electronic medical record. SSIs diagnosed after discharge were included when clinical documentation and microbiological confirmation were available within 30 days postoperatively.

### 4.6. Statistical Analysis

Statistical analysis was performed using SigmaXL v.11.03/2025 (SigmaXL Inc., Kitchener, ON, Canada). Descriptive statistics were used to summarize demographic, clinical, and microbiological data. Numerical data were expressed as frequency (number, *n*) and relative frequency (percentage, %) [65] (95%CI) [66].

Associations between categorical variables were assessed using the Chi-square test, with the R-squared (R-sq, coefficient of determination) and *p*-value calculated to identify statistically significant deviations from expected frequencies [67]. The correlations between patient age, IAI type, and antibiotics used in empirical therapy were investigated using the Chi-square test (Fisher’s Monte Carlo) and standardized Pearson residuals (RPS). A *p*-value < 0.05 was considered statistically significant.

## 5. Conclusions

The current study provides a detailed analysis of etiology, intra-abdominal infection type, susceptibility patterns, surgical intervention options, and age distribution among pediatric patients in a tertiary referral center. Overall, our findings indicate that broad-spectrum empirical therapy should not automatically lead to prolonged use of broad-spectrum antibiotics. Instead, empirical treatment should be quickly re-evaluated when microbiological results are available, enabling de-escalation to the narrowest effective agent. This approach improves clinical outcomes, while decreasing selective pressure for antimicrobial resistance and unnecessary carbapenem use. Moreover, the present work highlights the importance of ongoing collaboration between pediatric surgeons and microbiologists, to ensure optimal surgical management and appropriate antimicrobial therapy for IAIs in pediatric patients.

Future prospective multicenter studies, including the systematic identification of anaerobic pathogens, are necessary to refine empiric treatment strategies and improve pediatric outcomes. Furthermore, modifying the gut microbiota through probiotics, dietary interventions, or fecal transplantation may influence microbial composition and inflammation, potentially supporting empiric antimicrobial therapy in pediatric intra-abdominal infections, although current evidence specific to children remains limited.

## Figures and Tables

**Table 1:** Baseline data of pediatric patients.

Parameter	*n*	%	*n*	%	*n*	%	*p*-Value	R-sq (%)
	Total		Girls		Boys			
	177	100.00	64	36.16	113	63.84	ANOVA	
Age group (years)								
<1 year	12	6.78	4	6.25	8	7.08	0.29	13.99
1–3 years	21	11.86	8	12.50	13	11.50		
4–6 years	27	15.25	9	14.06	18	15.93		
7–10 years	39	22.03	17	26.56	22	19.47		
11–16 years	78	44.07	26	40.63	52	46.02		
IAI—type								
Primary IAI	170	96.05	62	96.88	108	95.58		
SSI	7	3.95	2	3.13	5	4.42		
Primary IAI								
Acute abdomen	1	0.59	0	0.00	1	0.93	0.36	2.08
Appendicitis (gangrenous)	23	13.53	7	11.29	16	14.81		
Appendicitis (gangrenous, with abscess)	1	0.59	0	0.00	1	0.93		
Appendicitis (gangrenous, with localized peritonitis)	5	2.94	0	0.00	5	4.63		
Appendicitis (perforated, with generalized peritonitis and abscesses)	1	0.59	0	0.00	1	0.93		
Appendicitis (perforated, with generalized peritonitis)	43	25.29	20	32.26	23	21.30		
Appendicitis (perforated, with localized peritonitis)	7	4.12	2	3.23	5	4.63		
Appendicitis (perforated)	3	1.76	2	3.23	1	0.93		
Appendicitis (perforated, with abscess)	4	2.35	3	4.84	1	0.93		
Appendicitis (phlegmonous)	4	2.35	2	3.23	2	1.85		
Bowel evisceration	2	1.18	2	3.23	0	0.00		
Intussusception	1	0.59	1	1.61	0	0.00		
Bowel occlusion	2	1.18	0	0.00	2	1.85		
Bowel perforation	3	1.76	0	0.00	3	2.78		
Bowel perforation with generalized peritonitis	1	0.59	0	0.00	1	0.93		
IA abscess	56	32.94	19	30.65	37	34.26		
Inguinal hernia	2	1.18	1	1.61	1	0.93		
Ovarian tumor	1	0.59	1	1.61	0	0.00		
Peritonitis	8	4.71	2	3.23	6	5.56		
Pyloric stenosis	1	0.59	0	0.00	1	0.93		
Small-bowel volvulus, mesenteric hernia	1	0.59	0	0.00	1	0.93		
SSI								
SSI after appendicitis (gangrenous) primary surgery	2	28.57	0	0.00	2	40.00	0.22	23.08
SSI after appendicitis (gangrenous, with generalized peritonitis) primary surgery	3	42.86	1	50.00	2	40.00		
SSI after inguinal lymph node excision	1	14.29	1	50.00	0	0.00		
SSI after peritonitis primary surgery (peritoneal abscesses)	1	14.29	0	0.00	1	20.00		

IAI = Intra-abdominal infection; SSI = surgical site infection; *n* = number (frequency); % = percentage (relative frequency); statistical tool: one-way ANOVA and means matrix; R-sq (%) = R-squared, coefficient of determination.

**Table 2:** Bacterial pathogens identified in pediatric patients’ specimens (expressed as percentages).

Aspect	1GN	1GP	1GN+1GP	1GN+2GP	2GN	2GN+1GP
	44.07	31.07	13.56	0.56	9.60	1.13
Bacterial species						
*C. freundii*	1.28	0.00	0.00	0.00	0.00	0.00
*C. freundii* + *Enterobacter* spp.	0.00	0.00	0.00	0.00	5.88	0.00
*C. freundii* + *Enterococcus* spp.	0.00	0.00	4.17	0.00	0.00	0.00
*E. coli* + *S. aureus*	0.00	0.00	8.33	0.00	0.00	0.00
*E. coli*	**83.33**	0.00	0.00	0.00	0.00	0.00
*E. coli* + *A. baumannii*	0.00	0.00	0.00	0.00	11.76	0.00
*E. coli* + *Enterococcus* spp.	0.00	0.00	20.83	0.00	0.00	0.00
*E. coli* + *Enterococcus* spp. + *Streptococcus* spp.	0.00	0.00	0.00	**100.00**	0.00	0.00
*E. coli* + *Klebsiella* spp.	0.00	0.00	0.00	0.00	17.65	0.00
*E. coli* + *P. aeruginosa*	0.00	0.00	0.00	0.00	**52.94**	0.00
*E. coli* + *P. aeruginosa* + *Streptococcus* spp.	0.00	0.00	0.00	0.00	0.00	**100.00**
*E. coli* + *Streptococcus* spp.	0.00	0.00	45.83	0.00	0.00	0.00
*Enterobacter* spp.	2.56	0.00	0.00	0.00	0.00	0.00
*Enterobacter* spp. + *Enterococcus* spp.	0.00	0.00	4.17	0.00	0.00	0.00
*Enterobacter* spp. + *Klebsiella* spp.	0.00	0.00	0.00	0.00	5.88	0.00
*Enterococcus* spp.	0.00	23.64	0.00	0.00	0.00	0.00
*Gemella morbillorum*	0.00	1.82	0.00	0.00	0.00	0.00
*H. alvei* + *Streptococcus* spp.	0.00	0.00	4.17	0.00	0.00	0.00
*Klebsiella* spp.	6.41	0.00	0.00	0.00	0.00	0.00
*Klebsiella* spp. + *Enterococcus* spp.	0.00	0.00	4.17	0.00	0.00	0.00
*Klebsiella* spp. + *P. aeruginosa*	0.00	0.00	0.00	0.00	5.88	0.00
*Klebsiella* spp. + *Streptococcus* spp.	0.00	0.00	4.17	0.00	0.00	0.00
*P. aeruginosa*	6.41	0.00	0.00	0.00	0.00	0.00
*P. aeruginosa* + *Enterococcus* spp.	0.00	0.00	4.17	0.00	0.00	0.00
*S. aureus*	0.00	**60.00**	0.00	0.00	0.00	0.00
*Streptococcus* spp.	0.00	14.55	0.00	0.00	0.00	0.00
Age group						
<1 year	7.69	9.09	0.00	0.00	5.88	0.00
1–3 years	12.82	12.73	8.33	0.00	11.76	0.00
4–6 years	12.82	16.36	20.83	**100.00**	5.88	**50.00**
7–10 years	21.79	10.91	29.17	0.00	**52.94**	0.00
11–16 years	44.87	**50.91**	41.67	0.00	23.53	**50.00**
IAI/SSI						
Acute abdomen	1.28	0.00	0.00	0.00	0.00	0.00
Appendicitis (gangrenous)	**29.49**	0.00	0.00	0.00	0.00	0.00
Appendicitis (gangrenous, with abscess)	1.28	0.00	0.00	0.00	0.00	0.00
Appendicitis (gangrenous, with localized peritonitis)	5.13	0.00	4.17	0.00	0.00	0.00
Appendicitis (perforated, with generalized peritonitis and abscesses)	1.28	0.00	0.00	0.00	0.00	0.00
Appendicitis (perforated, with generalized peritonitis)	**35.90**	3.64	**54.17**	0.00	0.00	0.00
Appendicitis (perforated, with localized peritonitis)	5.13	1.82	8.33	0.00	0.00	0.00
Appendicitis (perforated)	2.56	1.82	0.00	0.00	0.00	0.00
Appendicitis (perforated, with abscess)	2.56	1.82	4.17	0.00	0.00	0.00
Appendicitis (phlegmonous)	0.00	3.64	4.17	**100.00**	0.00	0.00
Bowel evisceration	2.56	0.00	0.00	0.00	0.00	0.00
Intussusception	0.00	1.82	0.00	0.00	0.00	0.00
Bowel occlusion	0.00	3.64	0.00	0.00	0.00	0.00
Bowel perforation	0.00	5.45	0.00	0.00	0.00	0.00
Bowel perforation with generalized peritonitis	0.00	1.82	0.00	0.00	0.00	0.00
IA abscess	**12.82**	**63.64**	**20.83**	0.00	**35.29**	0.00
Inguinal hernia	0.00	0.00	4.17	0.00	5.88	0.00
Ovarian tumor	0.00	0.00	0.00	0.00	5.88	0.00
Peritonitis	0.00	9.09	0.00	0.00	**11.76**	**50.00**
Pyloric stenosis	0.00	0.00	0.00	0.00	5.88	0.00
SSI after appendicitis (gangrenous) primary surgery	0.00	0.00	0.00	0.00	5.88	**50.00**
SSI after appendicitis (gangrenous, with generalized peritonitis) primary surgery	0.00	0.00	0.00	0.00	**17.65**	0.00
SSI after inguinal lymph node excision	0.00	1.82	0.00	0.00	0.00	0.00
SSI after peritonitis primary surgery (peritoneal abscesses)	0.00	0.00	0.00	0.00	5.88	0.00
Small-bowel volvulus, mesenteric hernia	0.00	0.00	0.00	0.00	5.88	0.00

Data are expressed as percentages (relative frequency); IAI—intra-abdominal infection; SSI—surgical site infection; GN = Gram-negative bacteria; GP = Gram-positive bacteria; 1GN = Gram-negative bacteria alone, 1GP = Gram-positive bacteria alone; 2GN = Two Gram-negative bacteria associated; 1GN+1GP = Dual association between 1 Gram-positive and 1 Gram-negative bacteria; 2GN+1GP and 2GP+1GN = Triple associations between Gram-positive and Gram-negative bacteria. Bolded values corresponding to “Bacterial species” and “Age groups” are ≥50%; bolded values related to “IAI/SSI” are >10%.

**Table 4:** Susceptibility patterns of *Enterobacter* spp. and *C. freundii*.

Antibiotic	Susceptibility							
	*n*	%	95%CI		*n*	%	95%CI	
	*Enterobacter* spp. (*n* = 2)				*C. freundii* (*n* = 1)			
Ertapenem	2	**100.00**	100.00	100.00	1	**100.00**	100.00	100.00
4th-gen. Cephalosporins	1	**50.00**	0.00	100.00	1	**100.00**	100.00	100.00
Piperacillin–Tazobactam	1	**50.00**	0.00	100.00	Not tested			
Ciprofloxacin	1	**50.00**	0.00	100.00				
Tetracycline	1	**50.00**	0.00	100.00	0	0.00	0.00	0.00
Monobactams	0	0.00	0.00	0.00	1	**100.00**	100.00	100.00
Trimetoprim–Sulfametoxazole	0	0.00	0.00	0.00	0	0.00	0.00	0.00
Amoxicillin–Clavulanic acid	0	0.00	0.00	0.00	0	0.00	0.00	0.00

Data is expressed as numbers and percentages; *n*—number (frequency); %—percentage (relative frequency). 95%CI = 95% confidence interval; 4th-gen. represents the generation of cephalosporins; fourth-generation cephalosporins are usually referred to as cefepime. Monobactams usually refer to Aztreonam. Bolded values are ≥50%.

**Table 5:** Gram-positive bacteria’s susceptibility patterns to various antibiotic drugs.

Antibiotic	*n*				Expected (%)	
	Total Tested	Susceptible	Resistant	Not Tested	Susceptibility	Resistance
***S. aureus* (*n* = 33)**						
Penicillin	33	0	33	0	0.00	**100.00**
Oxacillin	15	4	11	18	26.67	**73.33**
Ampicillin	1	0	1	32	0.00	**100.00**
Ampicillin–Sulbactam	1	0	1	32	0.00	**100.00**
Amoxicillin–Clavulanic acid	7	3	4	26	42.86	**57.14**
Aminoglycosides	29	29	0	4	**100.00**	0.00
Monobactams	1	1	0	32	**100.00**	0.00
Vancomycin	8	6	2	25	**75.00**	25.00
Linezolid	9	9	0	24	**100.00**	0.00
Teicoplanin	4	4	0	29	**100.00**	0.00
Clindamycin	25	14	11	8	**56.00**	44.00
Piperacillin–Tazobactam	2	1	1	31	50.00	50.00
Tetracycline	8	4	4	25	50.00	50.00
Trimetoprim–Sulfametoxazole	5	3	2	28	**60.00**	40.00
Fosfomycin	4	4	0	29	**100.00**	0.00
Fusidic acid	29	29	0	4	**100.00**	0.00
Macrolides	19	8	11	14	42.11	**57.89**
1st-gen. Cephalosporins	1	0	1	32	000	**100.00**
2nd-gen. Cephalosporins	1	0	1	32	0.00	**100.00**
4th-gen. Cephalosporins	1	1	0	32	**100.00**	0.00
3rd-gen. Cephalosporins						
Ceftazidime	1	0	1	32	0	**100.00**
Ceftriaxone	7	4	3	26	**57.14**	42.86
Carbapenems						
Meropenem	2	1	1	31	50.00	50.00
Imipenem	2	1	1	31	50.00	50.00
Fluoroquinolones						
Ciprofloxacin	20	14	6	13	**70.00**	30.00
Levofloxacin	3	2	1	30	**66.67**	33.33
Moxifloxacin	7	4	3	26	**57.14**	42.86
***Enterococcus* spp. (*n* = 13)**						
Ampicillin	6	2	4	7	33.33	**66.67**
Ampicillin–Sulbactam	1	1	0	12	**100.00**	0.00
Amoxicillin–Clavulanic acid	6	2	4	7	33.33	**66.67**
Aminoglycosides	8	7	1	5	**87.50**	12.50
Ciprofloxacin	13	8	5	0	**61.54**	38.46
Levofloxacin	1	1	0	12	**100.00**	0.00
Vancomycin	2	2	0	11	**100.00**	0.00
Linezolid	12	12	0	1	**100.00**	0.00
Teicoplanin	7	7	0	6	**100.00**	0.00
Tigecycline	7	7	0	6	**100.00**	0.00
Macrolides	6	6	0	7	**100.00**	0.00
Tetracycline	8	0	8	5	0.00	**100.00**
***Streptococcus* spp. (*n* = 8)**						
Penicillin	2	2	0	6	100.00	
Amoxicillin–Clavulanic acid	1	1	0	7	**100.00**	0.00
Linezolid	1	1	0	7	**100.00**	0.00
Teicoplanin	3	3	0	5	**100.00**	0.00
Clindamycin	2	2	0	6	**100.00**	0.00
Macrolides	4	4	0	4	**100.00**	0.00
Chloramfenicol	3	3	0	5	**100.00**	0.00
Tetracycline	1	1	0	7	**100.00**	0.00
2nd-gen. Cephalosporins	1	1	0	7	**100.00**	0.00
3rd Cephalosporins						
Ceftazidime	1	0	1	7	0.00	**100.00**
Ceftriaxone	7	7	0	1	**100.00**	0.00

Data are expressed as numbers (*n*) and percentages (%). Cephalosporins of the fourth generation usually refer to Cefepime. Monobactams usually refer to Aztreonam; Aminoglycosides usually refer to Amikacin. Macrolides usually refer to Azithromycin. 1st-, 2nd-, 3rd-, 4th-gen. represent the generation of cephalosporins. Bolded values from the Susceptibility and Resistance columns are ≥50%.

**Table 6:** IAIs/SSIs in pediatric patients of all age groups.

IAI/SSI	*n*	%	95%CI	
<1 year (*n* = 12)				
Bowel evisceration	1	8.33	0.00	23.97
Intussusception	1	8.33	0.00	23.97
IA abscess	9	**75.00**	50.50	99.50
Pyloric stenosis	1	8.33	0.00	23.97
1–3 years (*n* = 21)				
Acute abdomen	1	4.76	0.00	13.87
Appendicitis (gangrenous)	2	9.52	0.00	22.08
Appendicitis (gangrenous, with abscess)	1	4.76	0.00	13.87
Bowel evisceration	1	4.76	0.00	13.87
IA abscess	8	**38.10**	17.33	58.87
Inguinal hernia	1	4.76	0.00	13.87
Peritonitis	4	19.05	2.25	35.84
SSI after appendicitis (gangrenous, with generalized peritonitis) primary surgery	2	9.52	0.00	22.08
SSI after inguinal lymph node excision	1	4.76	0.00	13.87
4–6 years (*n* = 27)				
Appendicitis (gangrenous)	2	7.41	0.00	17.29
Appendicitis (perforated, with generalized peritonitis)	7	**25.93**	9.40	42.46
Appendicitis (perforated, with localized peritonitis)	4	14.81	1.42	28.21
Appendicitis (perforated, with abscess)	1	3.70	0.00	10.83
Appendicitis (phlegmonous)	1	3.70	0.00	10.83
Bowel occlusion	1	3.70	0.00	10.83
Bowel perforation	2	7.41	0.00	17.29
IA abscess	7	**25.93**	9.40	42.46
Peritonitis	2	7.41	0.00	17.29
7–10 years (*n* = 39)				
Appendicitis (gangrenous)	12	**30.77**	16.28	45.25
Appendicitis (gangrenous, with localized peritonitis)	2	5.13	0.00	12.05
Appendicitis (perforated, with generalized peritonitis)	11	**28.21**	14.08	42.33
Appendicitis (perforated, with localized peritonitis)	1	2.56	0.00	7.52
Appendicitis (perforated)	1	2.56	0.00	7.52
Appendicitis (perforated, with abscess)	1	2.56	0.00	7.52
Appendicitis (phlegmonous)	1	2.56	0.00	7.52
Bowel perforation with generalized peritonitis	1	2.56	0.00	7.52
IA abscess	5	12.82	2.33	23.31
Ovarian tumor	1	2.56	0.00	7.52
Peritonitis	1	2.56	0.00	7.52
SSI after appendicitis (gangrenous) primary surgery	1	2.56	0.00	7.52
SSI after peritonitis primary surgery (peritoneal abscesses)	1	2.56	0.00	7.52
11–16 years (*n* = 78)				
Appendicitis (gangrenous)	7	8.97	2.63	15.32
Appendicitis (gangrenous, with localized peritonitis)	3	3.85	0.00	8.11
Appendicitis (perforated, with generalized peritonitis and abscesses)	1	1.28	0.00	3.78
Appendicitis (perforated, with generalized peritonitis)	25	**32.05**	21.69	42.41
Appendicitis (perforated, with localized peritonitis)	2	2.56	0.00	6.07
Appendicitis (perforated)	2	2.56	0.00	6.07
Appendicitis (perforated, with abscess)	2	2.56	0.00	6.07
Appendicitis (phlegmonous)	2	2.56	0.00	6.07
Bowel occlusion	1	1.28	0.00	3.78
Bowel perforation	1	1.28	0.00	3.78
IA abscess	27	**34.62**	24.06	45.17
Inguinal hernia	1	1.28	0.00	3.78
Peritonitis	1	1.28	0.00	3.78
SSI after appendicitis (gangrenous) primary surgery	1	1.28	0.00	3.78
SSI after appendicitis (gangrenous, with generalized peritonitis) primary surgery	1	1.28	0.00	3.78
Small-bowel volvulus, mesenteric hernia	1	1.28	0.00	3.78

*n* = number (frequency); % = percentage (relative frequency); 95%CI = 95% confidence interval; bolded percentage values are >25%.

**Table 7:** Pathogenic bacteria distribution in pediatric patients of all age groups.

Bacteria	*n*	%	95%CI		Bacteria	*n*	%	95%CI	
**<1 year**					**1–3 years**				
1GN	6	**50.00**	21.71	78.29	1GN	10	**47.62**	26.26	68.98
1GP	5	**41.67**	13.77	69.56	1GP	7	**33.33**	13.17	53.50
1GN+1GP	0	0.00	0.00	0.00	1GN+1GP	2	9.52	0.00	22.08
1GN+2GP	0	0.00	0.00	0.00	1GN+2GP	0	0.00	0.00	0.00
2GN	1	8.33	0.00	23.97	2GN	2	9.52	0.00	22.08
2GN+1GP	0	0.00	0.00	0.00	2GN+1GP	0	0.00	0.00	0.00
**4–6 years**					**7–10 years**				
1GN	10	**37.04**	18.82	55.25	1GN	17	**43.59**	28.03	59.15
1GP	9	**33.33**	15.55	51.11	1GP	6	15.38	4.06	26.71
1GN+1GP	5	**18.52**	3.87	33.17	1GN+1GP	7	**17.95**	5.90	29.99
1GN+2GP	1	3.70	0.00	10.83	1GN+2GP	0	0.00	0.00	0.00
2GN	1	3.70	0.00	10.83	2GN	9	**23.08**	9.85	36.30
2GN+1GP	1	3.70	0.00	10.83	2GN+1GP	0	0.00	0.00	0.00
**11–16 years**									
1GN	35	**44.87**	33.83	55.91	1GN+2GP	0	0.00	0.00	0.00
1GP	28	**35.90**	25.25	46.54	2GN	4	5.13	0.23	10.02
1GN+1GP	10	**12.82**	5.40	20.24	2GN+1GP	1	1.28	0.00	3.78

*n* = number (frequency); % = percentage (relative frequency); 95%CI = 95% confidence interval; bolded percentage values are >30% for 1GP and 1GN and >10% for polymicrobial associations.

**Table 8:** Empirical antibiotherapy in 2GN-IAIs.

Antibiotic	Empirical Antibiotherapy								
	%	95%CI		%	95%CI		%	95%CI	
	***C. freundii* + *Enterobacter* spp.**			***E. coli* + *A. baumannii***			***E. coli* + *Klebsiella* spp.**		
Ampicillin–Sulbactam	0.00	0.00	0.00	**50.00**	0.00	100.00	0.00	0.00	0.00
Amoxicillin–Clavulanic acid	0.00	0.00	0.00	**50.00**	0.00	100.00	**66.67**	13.32	100.00
Aminoglycosides	0.00	0.00	0.00	**50.00**	0.00	100.00	0.00	0.00	0.00
1st-gen. Cephalosporins	0.00	0.00	0.00	**50.00**	0.00	100.00	33.33	0.00	86.68
4th-gen. Cephalosporins	**100.00**	100.00	100.00	**50.00**	0.00	100.00	**100.00**	100.00	100.00
Monobactams	**100.00**	100.00	100.00	0.00	0.00	0.00	**100.00**	100.00	100.00
Meropenem	0.00	0.00	0.00	0.00	0.00	0.00	0.00	0.00	0.00
Ertapenem	0.00	0.00	0.00	**50.00**	0.00	100.00	33.33	0.00	86.68
Ciprofloxacin	**100.00**	100.00	100.00	**50.00**	0.00	100.00	0.00	0.00	0.00
	***E. coli* + *P. aeruginosa***			***Enterobacter* spp. + *Klebsiella* spp.**			***Klebsiella* spp. + *P. aeruginosa***		
Amoxicillin–Clavulanic acid	**77.78**	50.62	100.00	0.00	0.00	0.00	0.00	0.00	0.00
Aminoglycosides	**100.00**	100.00	100.00	**100.00**	100.00	100.00	**100.00**	100.00	100.00
1st-gen. Cephalosporins	11.11	0.00	31.64	0.00	0.00	0.00	0.00	0.00	0.00
4th-gen. Cephalosporins	0.00	0.00	0.00	**100.00**	100.00	100.00	**100.00**	100.00	100.00
Monobactams	**100.00**	100.00	100.00	**100.00**	100.00	100.00	**100.00**	100.00	100.00
Meropenem	0.00	0.00	0.00	**100.00**	100.00	100.00	0.00	0.00	0.00
Ertapenem	0.00	0.00	0.00	**100.00**	100.00	100.00	0.00	0.00	0.00
Ciprofloxacin	0.00	0.00	0.00	**100.00**	100.00	100.00	0.00	0.00	0.00
Levofloxacin	0.00	0.00	0.00	**100.00**	100.00	100.00	0.00	0.00	0.00

Data are expressed as percentages. 95%CI = 95% confidence interval. Cephalosporins of the fourth generation usually refer to Cefepime. Monobactams usually refer to Aztreonam. Aminoglycosides usually refer to Amikacin. Bolded values are ≥50%.

**Table 9:** Empirical antibiotherapy in (1GN+1GP)-IAIs.

Antibiotic	Empirical Antibiotherapy								
	%	95%CI		%	95%CI		%	95%CI	
	***E. coli* + *Enterococcus* spp.**			***E. coli* + *S. aureus***			***E. coli* + *Streptococcus* spp**		
Penicillin	0.00	0.00	0.00	0.00	0.00	0.00	9.09	0.00	26.08
Ampicillin	0.00	0.00	0.00	**50.00**	0.00	100.00	0.00	0.00	0.00
Amoxicillin–Clavulanic acid	**80.00**	44.94	100.00	**50.00**	0.00	100.00	**81.82**	59.03	100.00
Aminoglycosides	20.00	0.00	55.06	**50.00**	0.00	100.00	9.09	0.00	26.08
Ceftriaxone	0.00	0.00	0.00	**50.00**	0.00	100.00	27.27	0.95	53.59
4th-gen. Cephalosporins	**80.00**	44.94	100.00	**100.00**	100.00	100.00	**50.00**	19.01	80.99
Piperacillin–Tazobactam	0.00	0.00	0.00	**50.00**	0.00	100.00	0.00	0.00	0.00
Monobactams	**60.00**	17.06	100.00	**100.00**	100.00	100.00	**72.73**	46.41	99.05
Meropenem	0.00	0.00	0.00	**50.00**	0.00	100.00	0.00	0.00	0.00
Ertapenem	20.00	0.00	55.06	**100.00**	100.00	100.00	0.00	0.00	0.00
Imipenem	0.00	0.00	0.00	**50.00**	0.00	100.00	0.00	0.00	0.00
Ciprofloxacin	40.00	0.00	82.94	0.00	0.00	0.00	0.00	0.00	0.00
Moxifloxacin	0.00	0.00	0.00	**50.00**	0.00	100.00	0.00	0.00	0.00
Vancomycin	0.00	0.00	0.00	**50.00**	0.00	100.00	0.00	0.00	0.00
Linezolid	0.00	0.00	0.00	**50.00**	0.00	100.00	0.00	0.00	0.00
Fusidic acid	0.00	0.00	0.00	**50.00**	0.00	100.00	0.00	0.00	0.00
Tigecyclin	0.00	0.00	0.00	**50.00**	0.00	100.00	0.00	0.00	0.00
Macrolides	0.00	0.00	0.00	0.00	0.00	0.00	30.00	1.60	58.40
Tetracycline	0.00	0.00	0.00	**50.00**	0.00	100.00	0.00	0.00	0.00
Chloramfenicol	0.00	0.00	0.00	0.00	0.00	0.00	18.18	0.00	40.97
Trimetoprim–Sulfametoxazole	0.00	0.00	0.00	**50.00**	0.00	100.00	0.00	0.00	0.00
	***Enterobacter* spp. +** ***Enterococcus* spp.**			***Klebsiella* spp. +** ***Enterococcus* spp.**			***P. aeruginosa* +** ***Enterococcus* spp.**		
Amoxicillin–Clavulanic acid	**100.00**	100.00	100.00	0.00	0.00	0.00	0.00	0.00	0.00
Aminoglycosides	0.00	0.00	0.00	0.00	0.00	0.00	**100.00**	100.00	100.00
4th-gen. Cephalosporins	0.00	0.00	0.00	**100.00**	100.00	100.00	0.00	0.00	0.00
Monobactams	0.00	0.00	0.00	**100.00**	100.00	100.00	0.00	0.00	0.00
Meropenem	0.00	0.00	0.00	**100.00**	100.00	100.00	0.00	0.00	0.00
Ertapenem	**100.00**	100.00	100.00	**100.00**	100.00	100.00	0.00	0.00	0.00
Ciprofloxacin	**100.00**	100.00	100.00	0.00	0.00	0.00	0.00	0.00	0.00
Levofloxacin	0.00	0.00	0.00	**100.00**	100.00	100.00	0.00	0.00	0.00
Colistin	0.00	0.00	0.00	0.00	0.00	0.00	**100.00**	100.00	100.00
Linezolid	0.00	0.00	0.00	0.00	0.00	0.00	**100.00**	100.00	100.00
Teicoplanin	0.00	0.00	0.00	**100.00**	100.00	100.00	0.00	0.00	0.00
Tigecyllin	0.00	0.00	0.00	**100.00**	100.00	100.00	0.00	0.00	0.00
Trimetoprim–Sulfametoxazole	0.00	0.00	0.00	**100.00**	100.00	100.00	0.00	0.00	0.00
	***C. freundii* + *Enterococcus* spp.**			***H. alvei* + *Streptococcus* spp.**			***Klebsiella* spp. + *Streptococcus* spp.**		
Amoxicillin–Clavulanic acid	0.00	0.00	0.00	0.00	0.00	0.00	**100.00**	100.00	100.00
4th-gen. Cephalosporins	**100.00**	100.00	100.00	**100.00**	100.00	100.00	**100.00**	100.00	100.00
Monobactams	**100.00**	100.00	100.00	**100.00**	100.00	100.00	0.00	0.00	0.00
Ertapenem	**100.00**	100.00	100.00	**100.00**	100.00	100.00	0.00	0.00	0.00
Ciprofloxacin	0.00	0.00	0.00	0.00	0.00	0.00	**100.00**	100.00	100.00

Data are expressed as percentages; 95%CI = 95% confidence interval. Cephalosporins of the fourth generation usually refer to Cefepime. Monobactams usually refer to Aztreonam. Aminoglycosides usually refer to Amikacin. Macrolides usually refer to Azithromycin. Bolded values are ≥50%.

## Data Availability

The data presented in this study are available on request from the first author and the first corresponding author, due to ethical reasons.

## Abbreviations

IAI	Intra-abdominal infection
cIAI	Complicated IAI
SSI	Surgical site infection
NEC	Necrotizing enterocolitis
CDC	Centers for Disease Control and Prevention
GN	Gram-negative
GP	Gram-positive
CT	Computer Tomography
PAD	Image-guided percutaneous drainage
EUCAST	European Committee on Antimicrobial Susceptibility Testing
IDSA	Infectious Diseases Society of America
ESBL	Extended-Spectrum Beta-Lactamase
SIS	Surgical Infections Society
RPS	Standardized Pearson residuals
R-sq	R-squared, coefficient of determination.
*n*	Number (frequency)
%	Relative frequency (percentage)
95%CI	95% Confidence Interval
CFU	Colony-forming units
Gen.	Generation (related to cephalosporins)
PAA	Post-Appendectomy Abscesses
IV	Intravenous
LOS	Length of stay in hospital
